# Association of Antenatal Risk Score With Maternal and Neonatal Mortality and Morbidity

**DOI:** 10.7759/cureus.12230

**Published:** 2020-12-22

**Authors:** Mohammed Y Al-Hindi, Thamer A Al Sayari, Raghad Al Solami, Anwar K AL Baiti, Jumanah A Alnemri, Iman M Mirza, Amjed Alattas, Yaser A Faden

**Affiliations:** 1 Pediatrics, King Abdulaziz Medical City - Western Region, Ministry of National Guard, Jeddah, SAU; 2 College of Medicine, King Saud Bin Abdulaziz University for Health Sciences, Jeddah, SAU; 3 Research and Development, King Abdullah International Medical Research Center- Western Region, Jeddah, SAU; 4 Medicine, Batterjee Medical College, Jeddah, SAU; 5 Medicine, Ibn Sina National College, Jeddah, SAU; 6 Community Medicine, University of Tabuk, Tabuk, SAU; 7 Pediatrics, King Abdullah Medical City, Jeddah, SAU; 8 Obstetrics and Gynecology, King Abdulaziz Medical City - Western Region, Ministry of National Guard, Jeddah, SAU; 9 Research and Development, King Abdullah International Medical Research Center - Western Region, Jeddah, SAU

**Keywords:** antenatal risk score, high-risk pregnancy, mortality, morbidity, maternal mortality, perinatal mortality, risk factors, prenatal care

## Abstract

Introduction/Objectives

Women with high-risk pregnancies require careful follow-up, management, and efficient allocation of resources to achieve optimal pregnancy outcomes. This study investigated the association between an updated, validated antenatal risk index score and neonatal mortality and morbidity in a tertiary care center in Saudi Arabia.

Methods

This retrospective cohort study included pregnant women delivered at King Abdulaziz Medical City, Jeddah, Saudi Arabia, between June 2016 and December 2018. Pregnant women who delivered before arrival, delivered in another hospital, or without an antenatal risk score because of missing data were excluded. The study cohort was recruited by simple random selection. Data of mothers and neonates were extracted from electronic health records. The pregnancy risk was assigned using a validated antenatal risk score index, creating low, moderate, and high-risk pregnancy categories. The association between antenatal risk scores, maternal and neonatal outcomes was investigated.

Results

A total of 533 pregnant women were included in the analysis, of whom 298 (55.9%) had low antenatal risk scores, 185 (34.7%) had moderate-risk scores, and 50 (9.4%) had high-risk scores. Maternal characteristics showed that high-risk mothers had higher age, gravidity, parity, and abortions than those with low or moderate-risk pregnancies. Newborns of high-risk mothers belonged more often to the male gender and had lower gestational ages, birth weights, and Apgar scores. For maternal outcomes, there was no maternal mortality. High-risk mothers had more cesarean sections and longer lengths of stay as compared to the low and moderate risk group. There was a trend toward increased stillbirths. Neonatal mortality, neonatal intensive care unit (NICU) admission, congenital anomalies, and length of stay were significantly increased in neonates of high-risk mothers.

Conclusions

An antenatal risk score is a feasible tool in identifying low, moderate, and high-risk pregnancies in a tertiary center outside a North American system. The higher scores were associated with maternal complications as well as neonatal mortality and morbidity. This is the first study to report maternal demographics, mortality, stillbirths, male gender, and congenital anomalies and their associations with categories of pregnancy level of risk. The clinical and economic benefits of antenatal risk screening in Saudi Arabia warrant further large population-based study that includes multi-domain socioeconomic determinants of health specific to our region.

## Introduction

The risk of maternal morbidity and mortality is of public concern worldwide. The World Health Organization estimates that about 830 women die daily from preventable causes related to pregnancy and childbirth [1]. High-risk pregnancies are those with factors associated with increased perinatal morbidity and mortality of pregnant women, fetuses, and neonates. Monitoring antenatal risk is a crucial element of routine care during pregnancy [1,2]. Antenatal risk scores are numerical indices that reflect the combined risk of existing maternal variables to identify women with high-risk pregnancies and prevent maternal and fetal complications. Goodwin et al. developed the first antenatal risk score in 1969, which was used to evaluate over 900 pregnancies in Toronto, Canada [3]. The system was validated by Burstyn [4]. The scores were categorized into low (0-2), moderate (3-6), and high (≥7) risk pregnancies. Burstyn found that most stillbirths and neonatal deaths were associated with high antenatal risk scores, increasing from 2.8/1000 live births for scores of 0-2 to 18.4/1000 live births for risk scores of 3-6. Unfortunately, maternal and neonatal mortality remains a significant health problem in Saudi Arabia and all developing countries [5]. To the best of our knowledge, no centers in Saudi Arabia are currently using antenatal risk scoring. Its applicability and reliability warrant further evaluation. The study aim was to determine whether there was an association between a validated antenatal risk index score and neonatal mortality and morbidity at a tertiary center in Jeddah, Saudi Arabia.

## Materials and methods

This is a retrospective study conducted by reviewing the medical records of all pregnant women who attended antenatal care and delivered at King Abdulaziz Medical City (KAMC), Jeddah, Saudi Arabia, between June 1, 2016, and December 31, 2018. Pregnant women who delivered before arrival, delivered in another hospital, or without an antenatal risk score because of missing data were excluded. Study subjects were included by simple random selection. Patient demographic data components and maternal and neonatal outcomes were extracted from electronic health records (Best-Care 2.0, http://www.ezcaretech.com/en/). The pregnancy risk was assigned using a validated antenatal risk score index that was adapted from Alberta perinatal health program and included low-, moderate-, and high-risk pregnancy categories [6]. The antenatal risk assessment score is based on 39 items that are used to classify each pregnant woman according to risks during pregnancy. A weighted value is assigned for each condition in the risk assessment tool, and the total score is the sum of these weighted values. Scores of 0 to 2 indicate lower risk, scores of 3 to 6 indicate moderate risk, and any risk score above 7 indicates a higher risk pregnancy. The antenatal risk score is composed of four parts. Part A includes a pre-pregnancy risk assessment that accounts for characteristics such as age, weight, and height as well as chronic diseases such as diabetes and hypertension. Part B includes obstetric history, including deaths, abortion, prematurity, cesarean section, data on whether babies were small or large for gestational dates, Rh isoimmunization, and major congenital anomalies. Part C describes current pregnancy problems, including data on whether fetuses are small or large for gestational age, amniotic fluid volume, multiple pregnancies, malpresentation, premature rupture of membranes, bleeding, gestational hypertension and diabetes, blood antibodies, anemia, post-term pregnancy, poor weight gain, and smoking status. Part D includes other risk factors such as major fetal anomalies, acute medical disorders, alcohol use, and drug dependence. The score from part D is yet to be validated, and hence, this score was not included in our study (Appendices). For additional details, the items and their corresponding scores are available from the Alberta Perinatal Health Program [6]. The Institutional Review Board of King Abdullah International Medical Research Center approved this study.

The estimated sample size was derived from the results of a study by Burstyn in which 10%, 21%, and 42% of the adverse outcomes occurred in low, moderate, and high-risk pregnancies, respectively [4]. Assuming that our population would have a similar distribution and assuming α = 0.05 and power (1β) = 80%, 405 patients would be required. The low- and moderate-risk groups required 166 each, and the high-risk group needed 73 each. To account for the possible low recruitment of women with high-risk pregnancies, the planned sample size was increased by 20% to include 486 patients.

The statistical analysis was performed by SPSS version 24 (IBM Corp., Armonk, NY, USA). Descriptive statistics included means and standard deviation with histograms to assess distribution and normality. Skewed data were reported as medians and interquartile range and as box plots. Categorical data were reported as proportions and bar charts. Inferential statistics for categorical outcomes included chi-square or Fisher exact tests and analysis of variance or Kruskal-Wallis tests for continuous variables depending on the data distribution.

## Results

**Figure 1:**
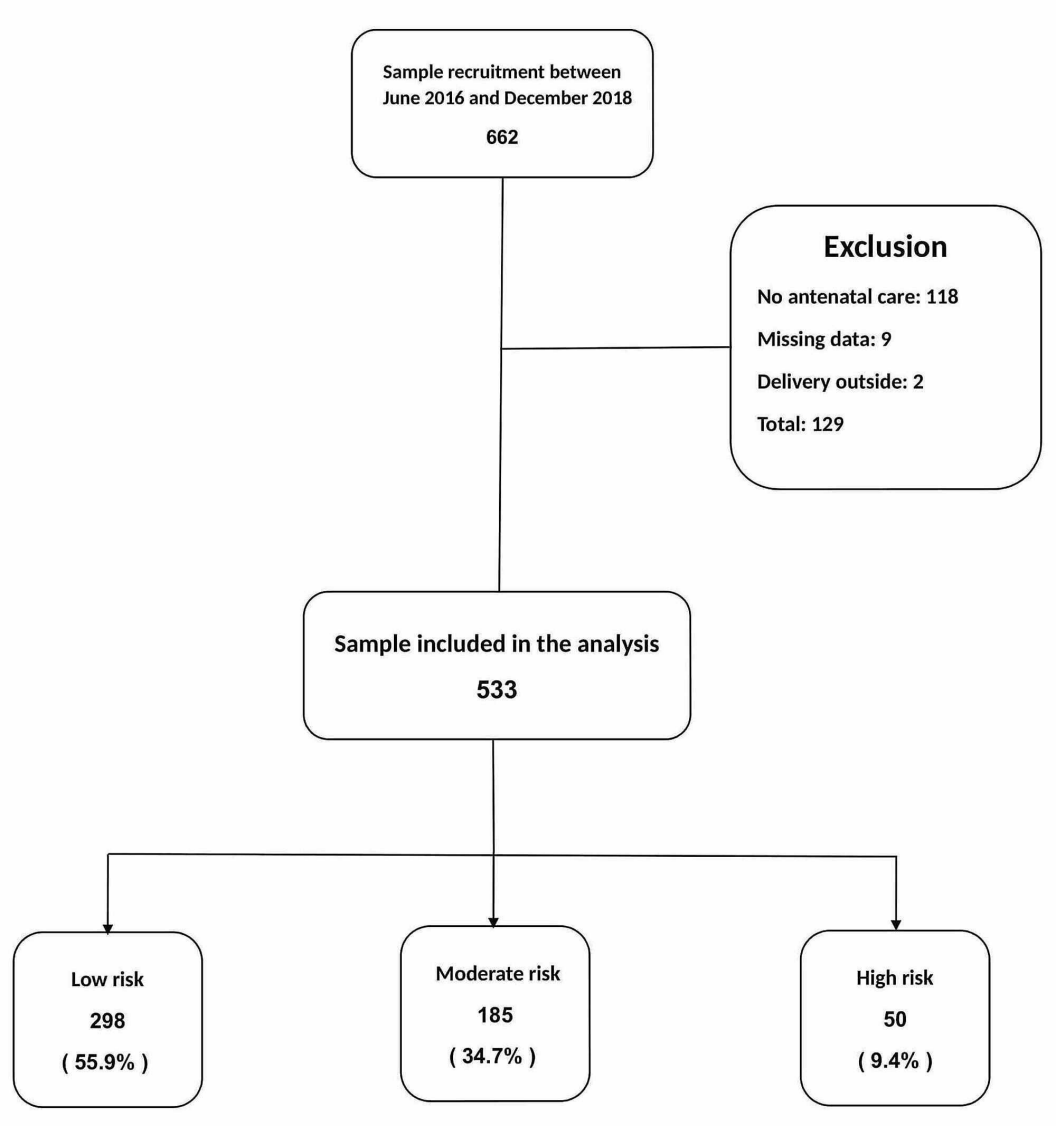
Patient flow

**Figure 2:**
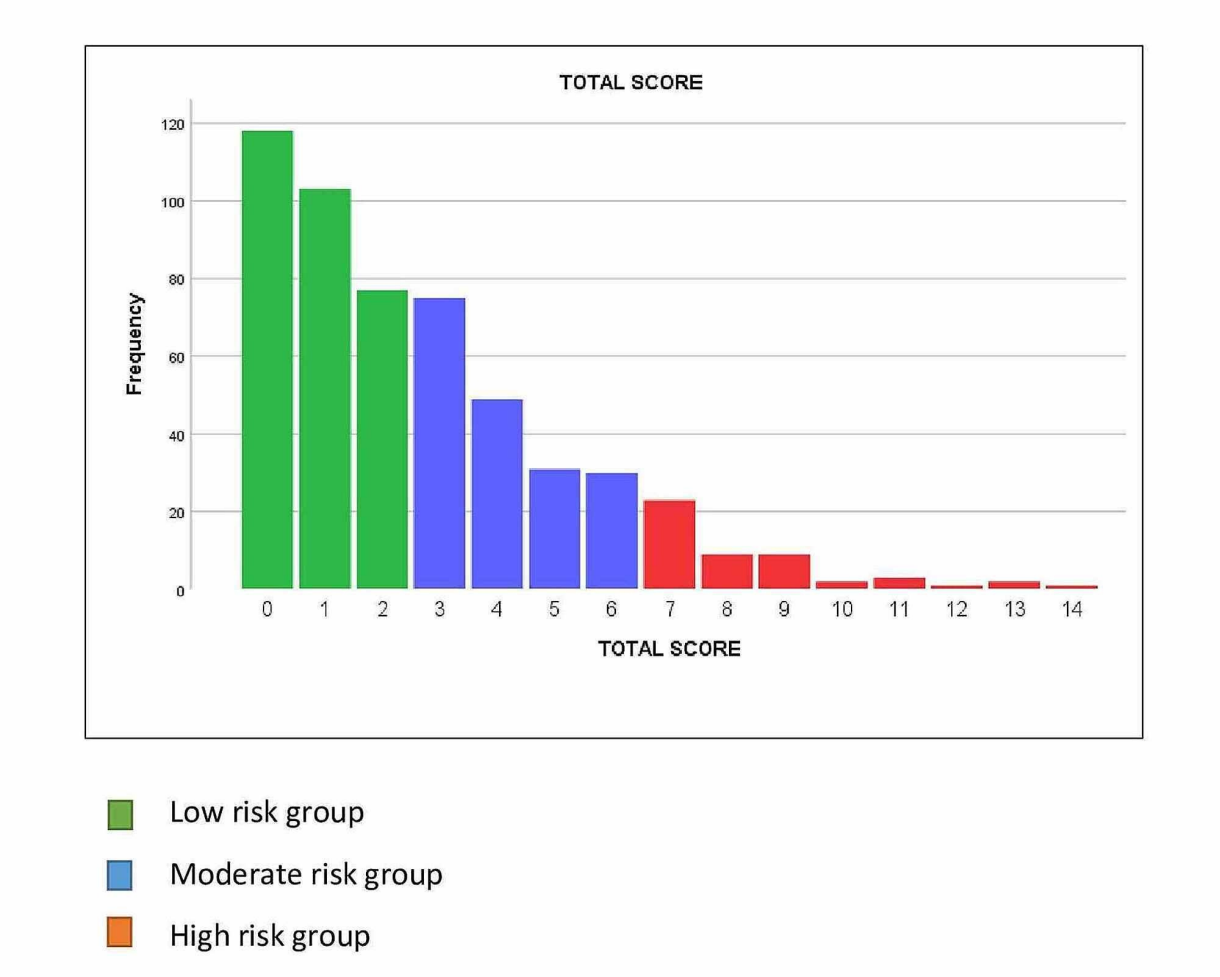
Antenatal risk score distribution

The patient disposition is shown in A total of 662 pregnant who received antenatal care or delivered at KAMC were recruited. Of these, 129 were excluded as per criteria, and the remaining 533 were included in the analysis. There were 298 (55.9%), 185 (34.7%), and 50 (9.4%) low-, moderate-, and high-risk pregnancies, respectively. The patients’ disposition is shown in Figure 1. The total scoring system was used in 533 mothers; the scores ranged from 0 to 14 with a median of 2 and a 50% interquartile range (1 to 4), as shown in Figure 2. The maternal and neonatal characteristics are shown in Table 1. As expected, as the pregnancy risk increased, a stepwise increase was observed in maternal age, gravidity, parity, and abortions, and these hikes were statistically significant. However, maternal body mass index (BMI) and group B Streptococcus (GBS) status were similar. As the risk of pregnancy increased, newborns had statistically significantly lower gestational age, birth weight, and Apgar scores at 5 minutes (Table 1). Males were more predominant in the high-risk pregnancy group. The maternal and neonatal outcomes are shown in Table 2. There was no maternal mortality in any risk group. Delivery was by cesarean section in 62%, 58.9%, and 20.5% of the high-, moderate-, and low-risk mothers (P < 0.001), respectively. High-risk mothers had an increased length of hospital stay: 3 (3-7) days versus 2 (2-3) days and 2 (2-2) days for moderate- and low-risk mothers (P < 0.001), respectively. There was a trend toward an increase in stillbirths in high-risk mothers; the rate was 80 per 1000 live births compared with 5.4 and 0 in mothers in the intermediate- and low-risk groups (P = 0.07), respectively. Maternal bleeding and admission to the ICU were higher in high-risk mothers; however, these differences were not statistically significant. On the contrary, instrumental delivery and maternal infection were similar among the groups. Neonatal mortality was 0.7%, 0.5%, and 6.2% for low-, moderate-, and high-risk pregnancies (P = 0.018), respectively. Neonatal intensive care unit (NICU) admissions, congenital anomalies, and length of stay were significantly increased in high-risk pregnancies, Table 2.

**Table 1:** Maternal–neonatal demographic *Mean (SD). **Median (50%IQR).

Maternal-neonatal demographic	Low risk (N=298)	Moderate risk (N=185)	High risk (N=50)	P-value
Maternal				
Maternal age*	28.7 (5.8)	30.5 (6.0)	34.1 (5.8)	<0.001
BMI*	30.5 (5.9)	30.8 (5.8)	31.9 (6.1)	0.3
Gravidity**	2 (1-4)	3 (2-5)	5 (3-7)	<0.001
Parity**	1 (0-2)	1 (0-3)	3 (0-5)	0.002
Abortion**	0 (0-0)	0 (0-1)	1 (0-2)	<0.001
GBS (% positive)	34.9%	37.4%	22.2%	0.32
Neonatal				
Birth weight (g)*	3049 (567)	2933 (676)	2472 (851)	<0.001
Gestational age* (weeks)	38.9 (1.8)	38 (2.6)	35.7 (4.0)	0.001
Gender (%male)	56.9%	44.9%	62%	0.015
5 minutes Apgar score (range)	9 (9-9)	9 (7-9)	9 (1-9)	<0.001

**Table 2:** Maternal–neonatal outcomes *Median (IQR). ^¥^Length of stay.

Maternal-neonatal outcomes	Low risk (N=298)	Moderate risk (N=185)	High risk (N=50)	P-value
Maternal				
Mortality	0	0	0	NA
Instrumental delivery	7.7%	5.4%	4%	0.5
Caesarian section	20.5%	58.9%	62%	<0.001
Bleeding	2.7%	2.7%	6%	0.4
Infection	2%	2.2%	2%	1
Admission to ICU	0.3%	0%	2%	0.1
Length of stay*^¥^	2 (2-3)	3 (2-4)	3 (3-4)	<0.001
Neonatal				
NICU admission	4.7%	14.1%	28%	<0.001
Congenital anomalies	3%	7.7%	14.3%	0.002
Mortality (per 1000 live birth)	0.7% (6.7)	0.5% (5.4)	6.2 (60)	0.018
Length of stay*	2 (2-2)	2 (2-3)	3 (2-7)	<0.001

## Discussion

This study showed that antenatal risk score application is useful and feasible in a tertiary care center outside the settings of North America. The distribution and the categorization of low (56% vs. 66%), moderate (34% vs. 29%), and high- (9.4% vs. 4.8%) risk pregnancies in this study was similar to that reported in a previous population-based study conducted in Canada except for the percentages of high-risk pregnancies [4]. The difference in high-risk pregnancies might be expected as KAMC is a tertiary referral center that accounted for 94% of perinatal deaths [2]. 

Similar to our study, an increased antenatal risk score was significantly associated with a poor outcome, but the scoring system was based on one that had been developed in 1977 and had not been validated by other studies [7]. The data had been collected at a single center in 1983-1984, and some records may have been incomplete because of paper-based reporting. Disease management and maternal demographics were both different at that time; some risk factors included in their scoring system were not included as ours, while others have updated definitions and management guidelines. We used an antenatal risk score adapted from Alberta perinatal health program [6] that was validated for neonatal morbidity and adverse pregnancy outcomes for singleton live births between 2001 and 2005 [4]. Our study can be considered as an updated validation of such scores in a different population.

Our study results are in line with those reported by Burstyn, with increases in stillbirths and neonatal deaths associated with increasing antenatal risk scores, 6.7 vs. 2.8/1000, and 10 vs. 18.4/1000 live births for low- and moderate-risk scores, respectively. The neonatal mortality associated with high-risk pregnancies in this study was 80/1000 live births [4]. As expected, neonatal mortality was the highest in high-risk pregnancies but cannot be directly compared across studies because of differences in the high-risk scoring [5]. Neonatal morbidities associated with high antenatal risk scores are difficult to characterize but appear across studies to be associated with gestational age, birth weight, five minutes Apgar score, and NICU admission [4,5]. In this study, the percentage of male births was more significant in high-risk pregnancies than in low- and moderate-risk pregnancies, which has not been reported before. Such an interesting association with fetal gender warrants a larger study to be validated. Moreover, this is the first study to report maternal demographics, mortality, and stillbirths in addition to congenital anomalies and their associations with categories of pregnancy level of risk.

Mothers who delivered before arrival were excluded from the analysis. Also, mothers with no antenatal care records, who comprised 17% of our sample, were excluded. This risk was not included in the Canadian antenatal score as a factor because it is rare to have pregnant women who are not followed up by healthcare professionals in the Canadian health care system [4]. Prospective studies of the value of antenatal score should be designed to include mothers with nil or less than four antenatal care visits as per WHO criteria [8], which comprise an important proportion in third world countries and Saudi Arabia.

Tertiary referral centers like ours are expected to experience more high-risk pregnancies. Even though the study results were consistent with those previously reported, this scoring system would benefit from validation in prospective studies at different hospital levels. The aim should be directed toward examining and improving healthcare systems, clinical pathways, and utilization of resources.

In this tertiary care center, many studies have investigated the effects of single maternal risk factors such as diabetes mellitus, antenatal coverage, small for gestational age, cesarean delivery, malpresentation, and body mass index on pregnancy outcomes [9-11]. However, the antenatal risk score, which is multifactorial, is not only an educational tool but also a comprehensive assessment of potential pregnancy complications. It is essential to evaluate each pregnancy to prevent maternal-fetal complications [12]. Notably, the antenatal risk score index is composed of biological and environmental factors and does not include the socioeconomic status of the mother. Socioeconomic status is composed of multiple domains, including education, occupation, and living conditions. The effects of socioeconomic status on pregnancy and newborns have been previously reported [13,14], and the inclusion of a multi-domain socioeconomic score warrants further study. Such determinants of health should be region-specific to account for our population characteristics.

This study is retrospective in nature and does not aim to prevent adverse pregnancy outcomes, but rather emphasizes the importance of careful follow-up, management, and reallocation of resources for pregnant women with high antenatal risk scores. The routine risk assessment will lead to optimal pregnancy outcomes by helping to identify the patients indicated for care in specialty centers and in planning safe delivery in local communities [12].

## Conclusions

An antenatal risk score is a feasible tool in identifying low, moderate, and high-risk pregnancies in a tertiary center outside a North American system. The higher scores were associated with maternal complications as well as neonatal mortality and morbidity. This is the first study to report maternal demographics, mortality, stillbirths, male gender, and congenital anomalies and their associations with categories of pregnancy level of risk. The clinical and economic benefits of antenatal risk screening in Saudi Arabia warrant further large population-based study that includes multi-domain socioeconomic determinants of health specific to our region.

## Appendices

**Table 3:** Antenatal risk assessment Antenatal risk assessment: Total the scores of parts A, B, and C. Low risk 0 - 2, moderate risk 3 - 6, and high risk > or = 7. The risk scores are cumulative and not exclusive of each other. N.B.: Adapted from the Alberta Perinatal Health Program, Suite 300 Kingsway Professional Centre, 10611 Kingsway Avenue, Edmonton, AB T5G 3C8 [5]. Part D was excluded as it was not validated.

Part A	Pre-pregnancy
Score	Condition
1	Age <17 at delivery
2	Age >35 at delivery
1	Weight >91 kg
1	Weight <45 kg
1	Height <152 cm
	Diabetes
1	Controlled by diet only
3	Insulin used
3	Retinopathy documented
	Heart disease
1	Asymptomatic (no effect on daily living)
3	Symptomatic (affects daily living)
	Hypertension
2	140/90 mmHg or greater
3	Antihypertensive drugs
2	Chronic renal disease documented
1	Other medical disorders, e.g., epilepsy, severe asthma, lupus, Crohn’s disease
Part B	Past obstetrical history
Score	Condition
3	Neonatal death(s)
3	Stillbirth(s)
1	Abortion between 12and 20 weeks and under 500 g of birth weight
1	Delivery at 20 - 37 weeks
2	Cesarean section
1	Small for dates - 5th percentile
1	Large for dates - 95th percentile
1	RH Iso-immunization - unaffected infant
3	RH Iso-immunization - affected infant
1	Significant congenital anomaly, e.g., Chromosomal, Heart, CNS defects
Part C	Problems in the current pregnancy
Score	Condition
2	Diagnosis of large for dates
3	Diagnosis of small for dates
2	Polyhydramnios or oligohydramnios
3	Multiple pregnancies
3	Malpresentation (breech or transverse lie)
2	Membranes ruptured before 37 weeks
1	Bleeding <20 weeks
3	Bleeding >20 weeks
2	Gestational hypertension
1	Proteinuria >1+
1	Gestational diabetes documented
3	Blood antibodies (Rh, Anti C, Anti K, etc.)
1	Anemia (Hgb <10 g/dL)
1	Pregnancy >41 weeks
1	Poor weight gain (26 - 36 weeks<0.5 kg/week or weight loss)
1	Smoker - anytime during pregnancy
	Total antepartum risk score

## References

[1] (2020). Maternal mortality. https://www.who.int/news-room/fact-sheets/detail/maternal-mortality.

[2] Ba’aqeel HS, Jabbar F, Al-Meshari AA (1989). Antenatal risk scoring form: statistical analysis of 1175 cases. Ann Saudi Med.

[3] Goodwin JW, Dunne JT, Thomas BW (1969). Antepartum identification of the fetus at risk. Can Med Assoc J.

[4] Burstyn I (2010). Antepartum risk score predicts adverse birth outcomes. J Obstet Gynaecol Can.

[5] Atasay B, Arsan S (2003). Organization of neonatal care services and its importance. J Perinat Med.

[6] (2020). Alberta Perinatal Health Program. Delivery record. https://www.yumpu.com/en/document/view/12005860/delivery-record-part-one-alberta-perinatal-health-program.

[7] Coopland AT, Peddle LJ, Baskett TF, Rollwagen R, Simpson A, Parker E (1977). A simplified antepartum high-risk pregnancy scoring form: statistical analysis of 5459 cases. Can Med Assoc J.

[8] Vogel JP, Habib NA, Souza JP (2013). Antenatal care packages with reduced visits and perinatal mortality: a secondary analysis of the WHO Antenatal Care Trial. Reprod Health.

[9] Ringholm L, Damm P, Mathiesen ER (2019). Improving pregnancy outcomes in women with diabetes mellitus: modern management. Nat Rev Endocrinol.

[10] Ekwempu CC (1988). The influence of antenatal care on pregnancy outcome. Trop J Obstet Gynaecol.

[11] Bodnar LM, Himes KP, Abrams B (2019). Gestational weight gain and adverse birth outcomes in twin pregnancies. Obstet Gynecol.

[12] Jain S, Anand S, Aherwar R (2014). High risk scoring for prediction of pregnancy outcome: a prospective study. Int J Reprod Contracept Obstet Gynecol.

[13] Al-Hindi MY, Aljuhani H, Alnajjar AR, Alessa S, Alqurashi M, Faden YA (2020). Examining the association between parental socioeconomic status and preterm birth using multidomain social determinants scale in a tertiary care center in Saudi Arabia. Cureus.

[14] Lefmann T, Combs-Orme T, Orme J (2017). Examining the inter-correlated effects of low income, life stress, and race on birth outcomes: a representative state study. Soc Work Health Care.

